# Genotype by environment interactions for chronic wasting disease in farmed US white-tailed deer

**DOI:** 10.1093/g3journal/jkac109

**Published:** 2022-05-10

**Authors:** Christopher M Seabury, Mitchell A Lockwood, Tracy A Nichols

**Affiliations:** Department of Veterinary Pathobiology, Texas A&M University, College Station, TX 77843, USA; Texas Parks and Wildlife Department, Austin, TX 78744, USA; USDA-APHIS-VS-Cervid Health Program, Fort Collins, CO 80526-8117, USA

**Keywords:** white-tailed deer, chronic wasting disease, GxE interaction, GWAA, *PRNP*

## Abstract

Despite implementation of enhanced management practices, chronic wasting disease in US white-tailed deer (*Odocoileus virginianus*) continues to expand geographically. Herein, we perform the largest genome-wide association analysis to date for chronic wasting disease (*n* = 412 chronic wasting disease-positive; *n* = 758 chronic wasting disease-nondetect) using a custom Affymetrix Axiom single-nucleotide polymorphism array (*n* = 121,010 single-nucleotide polymorphisms), and confirm that differential susceptibility to chronic wasting disease is a highly heritable ($h^{2}=\;0.611\;\pm \;0.056$) polygenic trait in farmed US white-tailed deer, but with greater trait complexity than previously appreciated. We also confirm *PRNP* codon 96 (G96S) as having the largest-effects on risk (*P *≤* *3.19E-08; phenotypic variance explained ≥ 0.025) across 3 US regions (Northeast, Midwest, South). However, 20 chronic wasting disease-positive white-tailed deer possessing codon 96SS genotypes were also observed, including one that was lymph node and obex positive. Beyond *PRNP*, we also detected 23 significant single-nucleotide polymorphisms (*P*-value ≤ 5E-05) implicating ≥24 positional candidate genes; many of which have been directly implicated in Parkinson’s, Alzheimer’s and prion diseases. Genotype-by-environment interaction genome-wide association analysis revealed a single-nucleotide polymorphism in the lysosomal enzyme gene *ARSB* as having the most significant regional heterogeneity of effects on chronic wasting disease (*P *≤* *3.20E-06); with increasing copy number of the minor allele increasing susceptibility to chronic wasting disease in the Northeast and Midwest; but with opposite effects in the South. In addition to *ARSB*, 38 significant genotype-by-environment single-nucleotide polymorphisms (*P*-value ≤ 5E-05) were also detected, thereby implicating ≥ 36 positional candidate genes; the majority of which have also been associated with aspects of Parkinson’s, Alzheimer’s, and prion diseases.

## Introduction

Chronic wasting disease (CWD) was initially recognized as a fatal wasting syndrome in captive mule deer (*Odocoileus hemionus*) and black-tailed deer *(Odocoileus hemionus columbianus*) housed within several Colorado wildlife research facilities during the late 1960s, with subsequent histological characterization as a prion disease by the late 1970s (Williams and Young 1980; Moreno and Telling 2018). Following initial characterization, CWD has subsequently been detected in free-ranging US elk (*Cervus elaphus nelsoni*), mule deer, white-tailed deer (*Odocoileus virginianus*; hereafter WTD), and moose (*Alces alces shirasi*), with further geographic expansion of the disease noted among farmed and free-ranging populations of these species (Moreno and Telling 2018; Gavin *et al.* 2019; Osterholm *et al.* 2019). Relevant to the geographic expansion of CWD, development and implementation of modern management practices, including the establishment of surveillance or containment zones, as well as depopulation of positive herds, have not prevented CWD from emerging in new geographic areas; with multiple Canadian provinces and at least 26 US states currently affected by CWD (Moreno and Telling 2018; Gavin *et al.* 2019; Osterholm *et al.* 2019). Moreover, Norway, Finland, and the Republic of Korea have also reported CWD in free-ranging reindeer (*Rangifer tarandus*; Norway), moose (*Alces alces*; Norway, Finland), and imported elk (Korea) (Moreno and Telling 2018; Gavin *et al.* 2019; Osterholm *et al.* 2019); thereby emphasizing the global expansion of CWD among several susceptible species within the family Cervidae. However, recent studies hypothesize that some forms of transmissible CWD may have emerged from sporadic CWD in Norwegian reindeer, moose, and perhaps red deer (*Cervus elaphus*) (Mysterud *et al.* 2020; Güere *et al.* 2021).

A recent genome-wide association study demonstrated that differential susceptibility to CWD, and natural variation in disease progression, are both moderately to highly heritable polygenic traits among farmed US WTD, and that loci other than *PRNP* are involved (Seabury *et al.* 2020). Moreover, genomic prediction accuracy related to differential susceptibility to CWD, as estimated by cross validation, was also shown to be high; thereby underscoring the potential for reducing susceptibility in farmed US WTD via genomic prediction (Seabury *et al.* 2020). However, no information currently exists regarding the potential for genotype-by-environment (GxE) interactions with respect to differential susceptibility to CWD. Herein, we employ genome-wide association analyses (GWAA) to further investigate the genomic basis for differential susceptibility to CWD in farmed US WTD using a larger and more geographically diverse sample than previously reported (Seabury *et al.* 2020), and subsequently confirm the high heritability of differential susceptibility to CWD. Additionally, we use two GWAA approaches to evaluate the potential for significant GxE interactions with respect to differential susceptibility to CWD among farmed US WTD. The results of this study provide the first genome-wide report on GxE interactions related to CWD, and are expected to positively augment genomic prediction programs aimed at reducing susceptibility among farmed US WTD.

## Materials and methods

### Study overview

In the present study, we utilize mixed linear models with genomic relationship matrices (GRM) to further investigate the genomic basis of differential susceptibility to CWD in farmed US WTD; including the potential for significant GxE interactions. Initially, we conduct standard GWAA with GRM heritability estimated using EMMAX (Kang *et al.* 2010; Segura *et al.* 2012), but also produce heritability estimates on the liability scale using GCTA (Lee *et al.* 2011; Yang *et al.* 2011) for 1,170 farmed US WTD. Thereafter, we use an implementation of EMMAX (Kang *et al.* 2010; Vilhjalmsson 2012; Smith *et al.* 2019) where interaction-term covariates may be specified; with the environmental variable expressing the US region of origin for each individual WTD (i.e. Northeast, Midwest, South) specified as the interaction term for a GxE GWAA. Finally, we also perform region-specific (i.e. Northeast, Midwest, South) GWAA for differential susceptibility to CWD using EMMAX (Kang *et al.* 2010; Segura *et al.* 2012), and thereafter, utilize a meta-based approach employing Cochran’s *Q*-test for heterogeneity (Cochran 1954; Willer *et al.* 2010) to further confirm WTD single-nucleotide polymorphisms (SNPs) displaying evidence for significant GxE interactions with respect to CWD.

### Animal resources, CWD diagnostics, and DNA isolation

Herein, we utilized animal repository resources, including CWD immunohistochemistry (IHC) diagnostic data (*n* = 523 CWD nondetect, *n* = 284 CWD positive), *PRNP* genotypes, and Affymetrix Axiom SNP array genotypes for 807 farmed US WTD from a previous study (Seabury *et al.* 2020). These data included regional representation from the US Northeast (*n* = 35), Midwest (*n* = 291), and South (*n* = 481), as previously described (Seabury *et al.* 2020). In the present study, we expand upon our prior dataset (*n* = 807) to collectively include 1,170 farmed US WTD from the Northeast (*n* = 286), the Midwest (*n* = 322), and the South (*n* = 562) by obtaining 363 additional farmed US WTD samples (both sexes) from an existing USDA APHIS repository that was created via federal CWD surveillance activities; including depopulations of CWD positive herds (USDA APHIS, Fort Collins, CO). Thus, the current dataset includes both CWD positive (*n* = 412) and CWD nondetect (*n* = 758) WTD (Thomsen *et al.* 2012) from 22 US farms with CWD prevalence ranging from 0.01 to 1.00 (Supplementary Table 1). For new WTD enrolled in the present study (*n* = 363), all CWD diagnostic classifications were based upon IHC (i.e. IHC of lymph node, obex, rectal, tonsil biopsy) that was performed at the USDA National Veterinary Services Laboratory (NVSL) in Ames Iowa (Thomsen *et al.* 2012). Genomic DNA for 363 additional farmed US WTD was isolated from ear fibroblast biopsies using the LGC sbeadex tissue purification kit (LGC) with automation at GeneSeek Neogen (Lincoln, NE) (Seabury *et al.* 2020). Thereafter, WTD genomic DNAs were quantified and assessed for purity (260/280 ratio) using a Nanodrop (ThermoFisher).

### *PRNP* and Affymetrix Axiom array genotyping

All *PRNP* genotyping (*n* = 363 farmed US WTD) for missense variants at codons 37, 95, 96, 116, and 226 was performed at GeneSeek Neogen (Lincoln, NE) via commercial genotyping by sequencing service (Seabury *et al.* 2020). Briefly, to prevent chimeric *PRNP* amplicons that obscure genotype–phenotype relationships, the functional *PRNP* gene was PCR amplified using primers exclusionary to a processed pseudogene (O’Rourke *et al.* 2004), with all amplicons purified via AMPure XP beads as recommended by the manufacturer (Beckman Coulter); thereby facilitating the generation of barcoded Illumina Nextera XT DNA libraries and amplicon sequencing on an Illumina MiSeq. All WTD *PRNP* genotypes for codons 37, 95, 96, 116, and 226 were called from the reference-aligned read pileups at GeneSeek Neogen, and delivered in text format (Seabury *et al.* 2020). Genotyping on the custom Affymetrix Axiom 200K SNP array for 363 farmed US WTD was also performed at GeneSeek Neogen using the Affymetrix best practices workflow; with genotypes delivered in text format (Seabury *et al.* 2020). Specifically, Affymetrix quality control thresholds implemented for the present study were DQC ≥ 0.82, QC call rate ≥95%, passing samples in the project ≥95%, and average call rate for passing samples ≥97%, as previously described and utilized (Seabury *et al.* 2020). For the present study, a total of 1,170 WTD samples passed all Affymetrix QC filters; each with 125,585 SNP array genotypes, and paired *PRNP* codon genotypes, thus yielding a combined set of 125,590 SNP genotypes for analysis. However, only 1,151 WTD samples contained all possible metadata (i.e. sex, age, US region of origin, farm code, CWD diagnostic outcome), and could be used for all analytical approaches explored in the present study.

### GWAA and GxE interactions

Because the draft *de novo* WTD genome assembly (GCF_002102435.1 Ovir.te_1.0) is unanchored (i.e. by maps or in situ hybridization), utilization of a comparative marker map (ARS-UCD1.2; GCA_002263795.2) is necessary to provide comparative evidence for the origin of the array and *PRNP* SNPs (i.e. autosomal *vs.* nonautosomal), as previously described (Seabury *et al.* 2020). After joining the comparative marker map to the combined set of all WTD genotypes (*PRNP* + Affymetrix Axiom array), quality control analyses were performed in SVS v8.9.0 (Golden Helix), including verification of sample call-rate (≥ 95%), and pairwise IBS distances to identify twins and duplicate samples among 1,170 US farmed WTD. No duplicate samples were detected. Further quality control analyses and filtering were as follows: SNP filtering by call rate (>15% missing excluded), MAF (< 0.01 excluded), polymorphism (monomorphic SNPs excluded), and Hardy–Weinberg Equilibrium (excludes SNPs with HWE *P*-value < 1e-25), thereby yielding 121,010 SNPs for all EMMAX GWAA involving 1,170 US farmed WTD. *PRNP* SNPs which failed to endure quality control filtering included only codon 116 (monomorphic), whereas codons 37, 95, 96, and 226 remained. To further investigate the genomic basis for differential susceptibility to CWD in farmed US WTD using a larger and more geographically diverse sample than previously reported, we performed GWAA on 1,170 WTD using a mixed linear model with variance component estimates, as implemented in EMMAX, and executed in SVS v8.9.0, with all genotypes recoded as 0, 1, or 2, based on the incidence of the minor allele (Kang *et al.* 2010; Segura *et al.* 2012; Neibergs *et al.* 2014; Seabury *et al.* 2017, 2020; Smith *et al.* 2019). However, within an additive model, hemizygous males may only possess 0 or 1 copy of an X-linked minor allele (excluding the pseudo-autosomal region), whereas females may possess 0, 1, or 2 copies of the minor allele. A gender correction reflecting these ploidy differences was utilized to recode putative X-linked genotypes prior to EMMAX-GRM analyses (Neibergs *et al.* 2014; Taylor 2014; Seabury *et al.* 2017, 2020). The disease phenotype used for all WTD analyses was CWD Binary (0 = nondetect, 1 = CWD positive for one or more diagnostic tissues including lymph node, obex, rectal, and/or tonsil). The mixed model utilized in the present study can be generally specified as: y=Xβ+Zu+ ϵ, where y represents a n×1 vector of CWD diagnostic phenotypes, X is a n×f matrix of fixed effects, β is a f×1 vector representing the coefficients of the fixed effects, u is the unknown random effect, and Z is a n×t matrix relating the random effect to the CWD diagnostic phenotypes (Kang *et al.* 2010; Segura *et al.* 2012; Seabury *et al.* 2017, 2020; Smith *et al.* 2019). Herein, we must assume that $\mathrm{Var}\left(u\right)=\sigma_{g}^{2}K$ and $\mathrm{Var}\left(\epsilon \right)=\sigma_{e}^{2}I$, such that $\mathrm{Var}\left(y\right)=\sigma_{g}^{2}ZKZ^{'}+\sigma_{e}^{2}I$, but in the present WTD study Z represents the identity matrix I, and K represents a relationship matrix of all WTD samples. To solve the mixed model equation using a generalized least squares approach, the variance components (i.e. $\sigma_{g}^{2}$ and $\sigma_{e}^{2}$) were estimated using the REML-based (restricted maximum likelihood) EMMA approach (Kang *et al.* 2010), with stratification accounted for and controlled using a GRM (G) (VanRaden 2008), as computed from the filtered WTD genotypes (*PRNP* + Affymetrix Axiom array) (Seabury *et al.* 2020). GRM heritability estimates ($\left[h^{2}=\;\sigma_{g}^{2}/(\sigma_{g}^{2}+\;\sigma_{e}^{2})\right]$) for differential susceptibility to CWD were produced as previously described (Kang *et al.* 2010; Segura *et al.* 2012; Seabury *et al.* 2017, 2020; Smith *et al.* 2019). Likewise, because the proportion of CWD cases included herein (i.e. 35% of 1,170 farmed WTD) is larger than the weighted mean CWD prevalence (i.e. 26%) across all farms included in the present study, we also estimated the heritability on a liability scale (Lee *et al.* 2011) using GCTA v1.93 (Yang *et al.* 2011) across a range of values for prevalence (i.e. prevalence = 0.01, 0.05, 0.10, 0.15, 0.20, 0.25, 0.26 as the weighted mean in the present study, 0.30, and 0.35). To conduct a GxE GWAA, we used the same filtered WTD data and disease phenotype (CWD Binary) in conjunction with an implementation of EMMAX (Kang *et al.* 2010; Vilhjalmsson 2012; Smith *et al.* 2019) whereby interaction-term covariates may be specified; with the environmental variable expressing the US geographic region of origin for each WTD (Northeast, Midwest, South) specified as the interaction term. The basis of this approach is rooted in full *vs.* reduced model regression (Neibergs *et al.* 2014; Smith *et al.* 2019), where interaction-term covariates are included in the model as follows: Each specified interaction-term covariate serves as one reduced-model covariate; Each specified interaction-term covariate is also multiplied, element by element, with each SNP predictor (i.e. SNP ×geographic origin) to create an interaction term to be included in the full model. Specifically, given n observations of a WTD disease phenotype (CWD Binary) that is influenced by m fixed effects and n instances of one random effect, with one or more GxE effects (e) whereby the interaction is potentially with one predictor variable, we model this using a full and a reduced model. The full model can be specified as $y\;=X_{c}\beta_{kc}+\;X_{i}\beta_{ki}+\;X_{k}\beta_{kp}+\;X_{ip}\beta_{ip}+u_{\mathrm{full}}+\;\epsilon_{\mathrm{full}}$, and the reduced model as $y\;=X_{c}\beta_{\mathrm{krc}}+\;X_{i}\beta_{\mathrm{kri}}+\;X_{k}\beta_{\mathrm{rkp}}+\;u_{\mathrm{reduced}}+\;\epsilon_{\mathrm{reduced}}$, where y is an n-vector of observed WTD CWD phenotypes, $X_{c}$ is an n ×m matrix of m fixed-effect covariates, $X_{i}$ is an n ×e matrix of e fixed terms being tested for GxE interactions,$\;X_{k}$ is an n-vector containing the covariate or predictor variable that may be interacting, and $X_{ip}$ is an n ×e matrix containing the e interaction terms created by multiplying the columns of $X_{i}$ element-by-element with $X_{k}$. Herein, all β terms correspond to the X terms as written above, and to the full or the reduced model, as specified, with u and ϵ representing the random effect and error terms, respectively (Smith *et al.* 2019). Similar to the EMMAX method without interactions (Kang *et al.* 2010; Segura *et al.* 2012), we approximate this by finding the variance components once, utilizing the parts of the above equations that are independent of $X_{k}$ as follows: $y\;=X_{c}\beta_{\mathrm{cvc}}+\;X_{i}\beta_{\mathrm{ivc}}+\;u_{vc}+\;\epsilon_{vc}$, where vc indicates the variance components. To estimate the variance components, we must again assume that $\mathrm{Var}\left(u_{vc}\right)=\sigma_{g}^{2}K$ and $\mathrm{Var}\left(\epsilon_{vc}\right)=\sigma_{e}^{2}I$, whereby Var$\left(y\right)=\sigma_{g}^{2}K+\;\sigma_{e}^{2}I$ (Kang *et al.* 2010; Vilhjalmsson 2012; Smith *et al.* 2019). The REML-based EMMA technique can then be used to estimate the variance components $\sigma_{g}^{2}$ and $\sigma_{e}^{2}$ as well as a matrix B (and its inverse) whereby $BB^{'}=H=\frac{\mathrm{Var}\left(y\right)}{\sigma_{g}^{2}}=\;K\;+\;\frac{\sigma_{e}^{2}}{\sigma_{g}^{2}}I$, as previously described and utilized in a large-sample analysis (Smith *et al.* 2019). Thereafter, for every WTD SNP marker (k), we can compute (via EMMAX-type approximation) the full and reduced models as:$\;B^{-1}y=\;B^{-1}X_{c}\beta_{kc}+\;B^{-1}X_{i}\beta_{ki}+\;{B^{-1}X}_{k}\beta_{kp}+B^{-1}X_{ip}\beta_{ip}+B^{-1}\left(u_{\mathrm{full}}+\;\epsilon_{\mathrm{full}}\right)$ for the full model, where $B^{-1}\left(u_{\mathrm{full}}+\;\epsilon_{\mathrm{full}}\right)$ is assumed to be an error term proportional to the identity matrix, and as $B^{-1}X_{c}\beta_{\mathrm{krc}}+\;B^{-1}X_{i}\beta_{\mathrm{kri}}+\;{B^{-1}X}_{k}\beta_{\mathrm{rkp}}+B^{-1}\left(u_{\mathrm{reduced}}+\;\epsilon_{\mathrm{reduced}}\right)$ for the reduced model, where$\;B^{-1}\left(u_{\mathrm{reduced}}+\;\epsilon_{\mathrm{reduced}}\right)$ is assumed to be an error term proportional to the identity matrix (Smith *et al.* 2019). To estimate the significance of the full *vs.* reduced model using the EMMAX GxE approach, an *F*-test was performed (Kang *et al.* 2010; Vilhjalmsson 2012; Smith *et al.* 2019); with all analyses executed and evaluated by constructing Manhattan plots within SVS v8.9.0 (Golden Helix, Bozeman, MT). Finally, although SVS computes the full model described above and outputs all β values, it only performs an optimization of the reduced model computation; to determine the residual sum of squares of the reduced-model equation, and thus estimate the full *vs.* reduced model *P*-value via *F*-test (Kang *et al.* 2010; Vilhjalmsson 2012). This general approach is highly efficient for large-sample analyses (Smith *et al.* 2019); with the reduced model optimization used to solve: ${MB}^{-1}y={MB}^{-1}X_{k}\beta_{\mathrm{rkp}}+\;\epsilon_{MB}$, where $M=\left(I-QQ^{'}\right)$, and Q is derived from performing the QR algorithm, as $QR=\;B^{-1}$[$X_{c}|X_{i}$]. Additional formulae and documentation are available at https://doc.goldenhelix.com/SVS/latest/svsmanual/mixedModelMethods/overview.html#gblupproblemstmt. Notably, because the probability of CWD infection is likely to increase with age (Grear *et al.* 2006), and may also disparately affect male and female WTD in different US regions, including differences in clinical disease progression and mortality (Grear *et al.* 2006; Edmunds *et al.* 2016), we explored several model fits for comparison as follows: GWAA with no fixed effect covariates; GWAA with sex, age, and US region of origin as fixed effect covariates; GWAA with sex, age, and US farm of origin (i.e. for farms with ≥10 deer available for analysis) as fixed effect covariates; GxE GWAA with no fixed effect covariates; GxE GWAA with sex and age as fixed effect covariates. A farm variable was not used as a fixed effect covariate for EMMAX GxE GWAA because farm and US region of origin are colinear. For all EMMAX analyses, genomic inflation factors were estimated in SVS v8.9.0 (Golden Helix) as: Pseudo-Lambda=log10(median observed *P*-value)/log10(median expected *P*-value).

For comparison to the EMMAX GxE approach (Kang *et al.* 2010; Vilhjalmsson 2012; Smith *et al.* 2019) utilizing 1,170 WTD, we also perform individual region-specific (i.e. Northeast, Midwest, South) GWAA for differential susceptibility to CWD using EMMAX (Kang *et al.* 2010; Segura *et al.* 2012), and thereafter, utilize a meta-based approach employing Cochran’s *Q*-test for heterogeneity of SNP effects (Cochran 1954; Willer *et al.* 2010). Briefly, SNP filtering for WTD from each US region (i.e. Northeast, Midwest, South), with additive recoding and gender correction, was performed as described above; thereby resulting in the following data sets for regional EMMAX GWAA: Northeast 124,977 SNPs (*n* = 116 CWD positive, *n* = 170 CWD nondetect); Midwest 125,446 SNPs (*n* = 208 CWD positive, *n* = 114 CWD nondetect); South 125,120 SNPs (*n* = 88 CWD positive, *n* = 474 CWD nondetect). Regional EMMAX GWAA were performed as described above within SVS 8.9.0 (Golden Helix) as follows: GWAA with no fixed effect covariates; GWAA with sex and age as fixed effect covariates; GWAA with sex, age, and US farm of origin as fixed effect covariates. The results of the regional EMMAX GWAA’s were utilized to conduct a sample-size (*Z*-score based) meta-analysis, as specified in the program METAL (Willer *et al.* 2010), and implemented in SVS 8.9.0 (Golden Helix). For every regional EMMAX GWAA, the SVS implementation of METAL (Willer *et al.* 2010) utilizes SNP marker *P*-values, the effect direction (SNP Predictor Beta), and sample sizes (for weighting purposes) to compute a *Z*-score and overall *P*-value, but also implements Cochran’s *Q*-test with *P*-values for identifying heterogeneity of SNP effects (Cochran 1954; Willer *et al.* 2010). Briefly, suppose that $N_{i}$ is the WTD sample size from study i, while $p_{ij}$ is the *P-*value from study i for SNP j, and $\Delta_{ij}$ is the direction of effect for study i at SNP j; the SVS v8.9.0 implementation of METAL (Willer *et al.* 2010) uses a normally distributed intermediate statistic $z_{ij}$, defined as $z_{ij}=\Phi^{-1}(p_{ij}/2)\mathrm{sign}(\Delta_{ij})$, to describe the effect, where $\Phi^{-1}$ denotes the inverse of 1 minus the cumulative distribution function of the normal distribution (the inverse survival function). Thereafter, using $w_{Zi}=\sqrt{N_{i}}$ to represent the *Z*-score weight for WTD study i, the overall *Z*-score for SNP j is computed as $Z_{j}=\frac{\sum_{i}z_{ij}w_{Zi}}{\sqrt{\sum_{i}w_{Zi}^{2}}}$, and the overall *P-*value is estimated as $P_{j}=2\Phi \left(\left|Z_{j}\right|\right),$ where Φ represents 1 minus the probability density function of the normal distribution (the survival function). Manhattan plots for the METAL-based meta-analysis (−log10 Overall *P*-value; −log10 Cochran’s Q *P*-value) were constructed and visualized in SVS v8.9.0 (Golden Helix). For all analyses (i.e. EMMAX, METAL), we employed a nominal significance threshold (*P*-value ≤5E-05) for polygenic traits (Wellcome Trust Case Control Consortium 2007; Neibergs *et al.* 2014; Seabury *et al.* 2017, 2020).

## Results and discussion

**Fig. 1.**
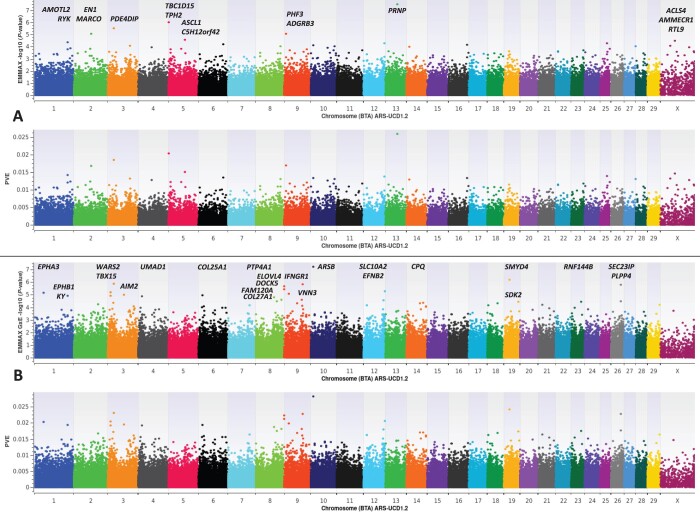
EMMAX binary case-control (0, 1) GWAA for CWD in farmed US white-tailed deer (*Odocoileus virginianus*; hereafter WTD). All dual-panel Manhattan plots depict −log10 *P*-values and the proportion of phenotypic variance explained (PVE) by white-tailed deer marker-effects on the *y*-axis, and the comparative position of all SNPs on the *x*-axis, as inferred by blastn alignment with the bovine genome (ARS-UCD1.2) (Seabury *et al.* 2020). All analyses include diagnostically confirmed CWD positive (*n* = 412) and CWD nondetect (*n* = 758) WTD. a) EMMAX GWAA for CWD with no fixed-effect covariates, high GRM heritability estimates ($h^{2}=\;0.611\;\pm \;0.056$) (Kang *et al.* 2010; Segura *et al.* 2012; Seabury *et al.* 2020), and relevant positional candidate genes. Genomic inflation factor (Pseudo-Lambda) = 1.007. b) EMMAX GxE GWAA for CWD with US WTD region of origin (Northeast, Midwest, South) as the environmental interaction term, and relevant positional candidate genes. Genomic inflation factor (Pseudo-Lambda) = 1.140.

A GWAA was conducted using a mixed linear model with GRM and variance component analysis, thereby producing a marker-based heritability estimate (GRM heritability) for differential susceptibility to CWD, as implemented in EMMAX (Kang *et al.* 2010; Segura *et al.* 2012), for a cohort of 1,170 farmed US WTD diagnostically classified (see *Materials and Methods*) as CWD positive (*n* = 412) and CWD nondetect (*n* = 758) from three US geographic regions (Northeast, Midwest, South). Notably, despite a 45% increase in overall sample size from our previous report (Seabury *et al.* 2020), including a more balanced sampling from each US geographic region (see *Materials and Methods*), the GRM heritability estimate remains comparatively high in this study (i.e. $h^{2}=\;0.611\;\pm \;0.056;\;\mathrm{previously}:\;h^{2}=\;0.637\;\pm \;0.070$); with the codon 96 missense variant (G96S) again displaying the largest genome-wide effects on differential susceptibility to CWD (Fig. 1, Supplementary Table 2). Likewise, heritability estimates on the liability scale (Lee *et al.* 2011; Yang *et al.* 2011) were also similarly high when CWD prevalence was ≥ 0.05 (i.e. $h^{2}\;\mathrm{from}\;\mathrm{Sum}\;\mathrm{of}\;V(G)\_L/Vp=\;0.557\;\pm \;0.053)$, and these estimates only increased with increasing CWD prevalence, thereby suggesting that our current and previous report (Seabury *et al.* 2020) likely provide conservative heritability estimates; particularly since the weighted mean CWD prevalence across all farms included in the present study was 0.26 (Supplementary Table 2). However, it is also interesting to note that given a much larger and more regionally diverse sample in this study, the proportion of phenotypic variance explained (PVE) by *PRNP* codon 96 is markedly lower (PVE ≤ 0.026) than previously reported (PVE ≤ 0.052) for 807 farmed US WTD (Seabury *et al.* 2020). Moreover, in the present study, we noted 20 CWD-positive WTD that possessed the codon 96SS genotype, including one that was both lymph node and obex positive. Collectively, for an EMMAX GWAA with 1,170 farmed US WTD, only eight SNPs met a nominal significance threshold (*P*-value ≤ 5E-05) for polygenic traits (Fig. 1, Supplementary Table 2) (Wellcome Trust Case Control Consortium 2007; Neibergs *et al.* 2014; Seabury *et al.* 2017, 2020), thereby confirming the CWD trait architecture previously described, where very few large or moderate-effect regions exist; but together with many small-effect regions, a significant proportion of the phenotypic variance can be explained (Seabury *et al.* 2020). Nevertheless, it should also be noted that EMMAX is known to produce conservative *P*-values (Zhou and Stephens 2012). In addition to *PRNP*, an investigation of nominally significant SNPs (*P*-value ≤ 5E-05) revealed positional candidate genes previously implicated in aspects of prion disease (*TPH2*; *PDE4DIP*), including scrapie (*ACSL4*), regulation of the central nervous system (*ADGRB3*), neuroprotection (*EN1*), Alzheimer’s (*ASCL1*, *AMOTL2*, *RYK*), and Parkinson’s disease (*EN1*, *ASCL1*, *RTL9*) (Ide *et al.* 2005; Roffé *et al.* 2010; Filali *et al.* 2014; Nishizawa *et al.* 2014; Alleaume-Butaux *et al.* 2015; Rekaik *et al.* 2015; Dunn *et al.* 2019; Meyer *et al.* 2019; Scuderi *et al.* 2019; Feng et al. 2020; Gallart-Palau *et al.* 2020; Le Guen et al. 2020). Additional missense variants encoded by *PRNP* codons 37, 95, and 226 did not meet the nominal significance level (*P*-value ≤ 5E-05) for polygenic traits (Wellcome Trust Case Control Consortium 2007; Neibergs *et al.* 2014; Seabury *et al.* 2017, 2020). Importantly, EMMAX mixed model solutions for the binary CWD case-control trait were robust to the inclusion of additional fixed effect covariates (i.e. sex, age, US. region of origin; and/or sex, age, farm); as the majority of the significant SNPs (*P*-value ≤ 5E-05) detected were shared across all analyses, including *PRNP* codon 96, which consistently displayed the largest genome-wide effects on differential susceptibility to CWD (Fig. 1, Supplementary Table 2 and Supplementary Fig. 1). Detailed summary data for all EMMAX GWAA’s, including PVE, the direction of all SNP effects, Supplementary Manhattan plots, genomic inflation factors (Pseudo-Lambda), and *PP*-Plots are provided in Additional Files 1–7 in DRYAD (https://doi.org/10.5061/dryad.wh70rxwnt).

To investigate the potential for significant GxE interactions with respect to differential susceptibility to CWD, we conducted a GxE GWAA using EMMAX (see *Materials andMethods*). Collectively, 27 SNPs met the nominal significance level (*P*-value ≤ 5E-05) for polygenic traits (Wellcome Trust Case Control Consortium 2007; Neibergs *et al.* 2014; Seabury *et al.* 2017, 2020), with no significant SNPs noted within or proximal to *PRNP* (Fig 1., Supplementary Table 2). Notably, the largest-effect GxE signal detected for differential susceptibility to CWD was in *ARSB* (intron 4); a gene that encodes a lysosomal enzyme (Arylsulfatase B) required for the catabolism of glycosaminoglycans (GAGs), including N-acetyl-d-galactosamine, dermatan sulfate, and chondroitin sulfate. Mutations in *ARSB* that result in a defective protein (i.e. enzyme deficiency) are known to be causal for the lysosomal storage disease known as Mucopolysaccharidosis (MPS) Type VI; with the concentration of urinary GAGs generally presenting as 5-100 times higher in patients with various forms of MPS (Pastores and Maegawa 2013; Sun *et al.* 2015; Vairo *et al.* 2015; Malekpour *et al.* 2018; Wang *et al.* 2018). Interestingly, the metabolism of GAGs is also known to be impaired in both humans and animals suffering from prion disease; with the degradation of GAGs disrupted by their interaction with PrP^Sc^, thus resulting in their accumulation and secretion in urine (Mayer-Sonnenfeld *et al.* 2005). Moreover, hexosaminidase is known to be one of the last enzymes functioning in the degradation cascade for several GAGs (i.e. chondroitin sulfate, dermatan sulfate, and keratan sulfate), and its enzymatic activity is significantly elevated in the brains of scrapie-infected mice, as compared to controls (Mayer-Sonnenfeld *et al.* 2005). However, the relationship between GAGs and prion diseases in humans and animals is somewhat complex, as the presence of GAGs (i.e. heparan sulfate; chondroitin sulfate) enhances PrP^Sc^ biogenesis and accumulation in cells, but the opposite has also been well postulated; where the accumulation of PrP^Sc^ may somehow cause an increase in GAG accumulation, particularly in lysosomes (Ben-Zaken *et al.* 2003; Mayer-Sonnenfeld *et al.* 2005). To the authors’ best knowledge, this is the first report to ever demonstrate a direct genetic association between a lysosomal enzyme gene involved in GAG catabolism (i.e. dermatan sulfate; chondroitin sulfate), and prion disease (CWD); yet the presence of weakly and strongly sulfated GAGs (i.e. chondroitin, heparan, karatan, and/or heparin) have been known to colocalize with amyloid plaques in CWD-affected captive mule deer for more than 30 years (Guiroy *et al.* 1991). However, amyloid plaques were not uniformly found in all CWD positive mule deer (Guiroy *et al.* 1991). Notably, a more recent study in mice demonstrates that MPS can lead to amyloidosis, synucleinopathy, and an apparent prion encephalopathy; with the accumulation of misfolded proteins generally considered to be an indirect result of progressive failure of lysosomal function in inbred mice (Naughton *et al.* 2013). Therefore, this raises the possibility that CWD may potentially present diagnostically in the absence of an infectious exposure (i.e. sporadically), and that future research should focus on the pathophysiological timing and potentially complex biochemical mechanisms of disease, as well as variation in PrP^CWD^ trafficking, including quantification of live-animal shedding given different genomic backgrounds (Seabury *et al.* 2020). Interestingly, in the present study, the *ARSB* SNP displaying significant GxE effects was observed to increase susceptibility to CWD in both the Northeast and the Midwest, but had the opposite direction of effect in the South; thereby underscoring the overall trait complexity. Beyond *ARSB* and its association with MPS, we also noted 24 positional candidate genes related to 26 additional EMMAX GxE signals (*P*-value ≤ 5E-05; Supplementary Table 2, Fig. 1); the majority of which have previously been associated with Parkinson’s disease (*SMYD4*, *WARS2*, *IFNGR1*, *PLPP4*, *ASCL1*, *FAM120A*), Alzheimer’s disease (*TBX15*, *IFNGR*, *PTP4A1*, *AIM2*, *SLC10A2*, *COL25A1*, *ASCL1*, *EPHB1*, *UMAD1*, *VNN3*, *COL27A1*, *RNF144B*, *SDK2*), and various prion diseases (*IFNGR*, *SEC23IP*, *EPHA3*, *EFNB2*, *ELOVL4*, *DOCK5*, *COL27A1*) including scrapie, bovine spongiform encephalopathy, and Creutzfeldt–Jakob disease (Ide *et al.* 2005; Julius 2008; Hashioka *et al.* 2009; Tong *et al.* 2010; Tian *et al.* 2013; Woodling *et al.* 2014; Majer 2015; Freeman and Ting 2016; Vélez *et al.* 2016; Watson *et al.* 2016; Mez *et al.* 2017; Su *et al.* 2018; Choubey 2019; Dabin 2019; Hirsch *et al.* 2019; Liu *et al.* 2019; Majer *et al.* 2019; Meyer *et al.* 2019; Thatra 2019; Bellenguez et al. 2020; Dabin *et al.* 2020; Donaldson *et al.* 2020; Martinelli *et al.* 2020; Wang *et al.* 2020; Vastrad and Vastrad 2021). Notably, the EMMAX GxE mixed model solutions were also robust to the inclusion of additional fixed effect covariates (i.e. sex, age; Supplementary Table 2 and Supplementary Fig. 2); as the majority of the significant SNPs (*P*-value ≤ 5E-05) detected were shared across all analyses, including *ARSB*, which consistently displayed the most significant genome-wide GxE interactions related to CWD susceptibility. Detailed summary data for all EMMAX GxE GWAA’s, including SNP-based regional interactions and directions of effect, a Supplementary Manhattan plot (Supplementary Fig. 2), genomic inflation factors (Pseudo-Lambda), and *PP*-Plots are provided in Additional Files 8–12 in DRYAD (https://doi.org/10.5061/dryad.wh70rxwnt).

**Fig. 2.**
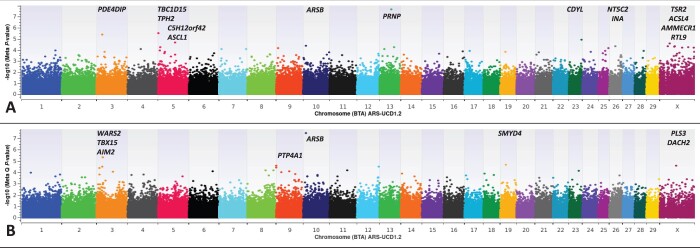
Binary case-control (0, 1) meta-analysis for differential susceptibility to CWD in farmed US white-tailed deer (*Odocoileus virginianus*; hereafter WTD) from the Northeast, Midwest, and South. Individual EMMAX GWAA’s (Kang *et al.* 2010; Segura *et al.* 2012; Seabury *et al.* 2020) for each US region were used in conjunction with the METAL-based approach to conduct a meta-analysis (Willer *et al.* 2010). METAL-based analyses included diagnostically confirmed CWD positive (*n* = 412) and CWD nondetect (*n* = 758) WTD. a) METAL-based *Z*-score analysis of shared WTD SNP effects and positional candidate genes influencing differential susceptibility to CWD across 3 US regions (Northeast, Midwest, South). Genomic inflation factor (Pseudo-Lambda) = 1.015. b) METAL-based Cochran’s *Q*-test for heterogeneity of SNP effects (Cochran 1954; Willer *et al.* 2010) across 3 US regions (Northeast, Midwest, South) and relevant positional candidate genes. Genomic inflation factor (Pseudo-Lambda) = 0.997.

For comparison to our EMMAX GxE analysis with WTD region of origin as the interaction term, we performed individual EMMAX GWAA’s for each US region (Northeast, Midwest, South), and used the corresponding regional results (Additional Files 13–15 in DRYAD; (https://doi.org/10.5061/dryad.wh70rxwnt) to conduct a meta-analysis, as previously described and implemented in the program METAL (Willer *et al.* 2010). Collectively, the majority of the significant EMMAX main effect SNPs were also detected by METAL (*P*-value ≤ 5E-05); with the codon 96 missense variant (G96S) displaying the most significant genome-wide effects on differential susceptibility to CWD across all US regions (Fig. 2, Supplementary Table 2). However, the METAL-based approach also identified a significant main-effect SNP in *ARSB* (intron 6) which was not significant by EMMAX GWAA (Fig. 1, Supplementary Table 2), but nonetheless, was among the top 13 ranked SNPs (Additional Files 1–4 in DRYAD: (https://doi.org/10.5061/dryad.wh70rxwnt). Five additional main-effect SNPs not detected by EMMAX were also detected by METAL; with positional candidate genes previously associated with Parkinson’s disease (*CDYL*, *NT5C2*), Alzheimer’s disease (*CDYL*), pathological inclusions of neuronal intermediate filaments (*INA*), and scrapie (*NT5C2*, *TSR2*) (Cairns *et al.* 2004; Filali *et al.* 2014; Nalls *et al.* 2014; Majer 2015; Lo *et al.* 2020; Aslam *et al.* 2021). Relevant to our EMMAX GxE analysis, SNPs displaying evidence of significant heterogeneity of effects, as evidenced by Cochran’s *Q*-test, included *ARSB* (intron 4) as the most significant GxE interaction with respect to differential susceptibility to CWD (Fig. 2, Supplementary Table 2). In addition to *ARSB*, METAL-based analysis also identified eight additional SNPs with significant heterogeneity of effects across US regions; seven of which were also detected by EMMAX GxE GWAA (Fig. 2, Supplementary Table 2). One significant SNP that was detected in our METAL-based analysis via Cochran’s *Q*-test for heterogeneity was intergenic between *PLS3* and *DACH2*; with *DACH2* previously implicated in the pathophysiology of scrapie (Gossner and Hopkins 2015). However, it should also be noted that the same SNP implicating *DACH2* was also among the top 39 ranked SNPs in a EMMAX GWAA (Fig. 1, Additional File 1), and the most significant SNP in a regional EMMAX GWAA for farmed WTD in the US South (Additional File 15; DRYAD: https://doi.org/10.5061/dryad.wh70rxwnt). Altogether, these results are intriguing considering that the molecular phenotype of experimentally passaged CWD in sheep is known to be indistinguishable from some strains of scrapie in sheep (Cassmann *et al*. 2021). Application of the METAL-based meta-analysis approach to regional EMMAX GWAA’s with and without additional fixed effect covariates (i.e. sex and age; sex, age, and farm) demonstrated that the majority of the significant main effect SNPs, and those displaying significant heterogeneity of effects across three US regions, were shared across all analyses. Thus, the mixed model solutions for various US regional model fits consistently implicate an overlapping set of the same significant SNPs and corresponding positional candidate genes. Detailed summary data for all METAL-based meta-analyses, including the EMMAX mixed model solutions from all regional model fits, and all corresponding METAL-based meta-analysis results with *PP*-Plots are provided in Additional Files 13–30 in DRYAD (https://doi.org/10.5061/dryad.wh70rxwnt). Collectively, our analyses of these data are compatible with several prior studies; where aspects of prion disease presentation were largely influenced by a genetic architecture independent of *PRNP* (Kingsbury *et al.* 1983; Stephenson *et al.* 2000; Iyegbe *et al.* 2010; Seabury *et al.* 2020).

## Conclusions

Herein, we perform the largest GWAA to date for CWD in WTD, thereby further confirming that differential susceptibility to CWD is a highly heritable, polygenic trait in farmed US WTD, but with greater overall complexity than previously postulated or reported; as evidenced by significant GxE interactions, the general paucity of moderate or large-effect SNPs, and conversely, the large number of SNPs displaying small effects on risk. We also confirm *PRNP* codon 96 as the largest-effect region of the WTD genome across 3 US regions (Northeast, Midwest, South). However, the proportion of phenotypic variance explained (PVE) by *PRNP* SNPs alone cannot be expected to facilitate a successful CWD eradication program, as further evidenced by 20 CWD positive WTD possessing the codon 96SS genotype enrolled in the present study; including one that was both lymph node and obex positive. Finally, we provide the first evidence linking naturally occurring genetic variation in a lysosomal GAG catabolism gene (*ARSB*) to differences in CWD susceptibility in farmed US WTD, but also further confirm the involvement of genes underlying other neurodegenerative diseases such as Parkinson’s, Alzheimer’s, and various prion diseases of mammals, including scrapie and sporadic Creutzfeldt–Jakob disease.

## Data availability

Accession codes are as follows: Data (DRYAD: https://doi.org/10.5061/dryad.wh70rxwnt).

Supplemental material is available at *G3* online. 

## Supplementary Material

jkac109_Supplementary_Table_S1

jkac109_Supplementary_Table_S2
